# Allele-specific regulation of *FGFR2 *expression is cell type-dependent and may increase breast cancer risk through a paracrine stimulus involving *FGF10*

**DOI:** 10.1186/bcr2917

**Published:** 2011-07-18

**Authors:** Petra EA Huijts, Minka van Dongen, Moniek CM de Goeij, Adrian J van Moolenbroek, Freek Blanken, Maaike PG Vreeswijk, Esther M de Kruijf, Wilma E Mesker, Erik W van Zwet, Rob AEM Tollenaar, Vincent THBM Smit, Christi J van Asperen, Peter Devilee

**Affiliations:** 1 Department of Clinical Genetics, Leiden University Medical Center, PO Box 9600, 2300 RC Leiden, The Netherlands; 2 Department of Human Genetics, Leiden University Medical Center, PO Box 9600, 2300 RC Leiden, The Netherlands; 3 Department of Surgery, Leiden University Medical Center, PO Box 9600, 2300 RC Leiden, The Netherlands; 4 Department of Medical Statistics and Bioinformatics, Leiden University Medical Center, PO Box 9600, 2300 RC Leiden, The Netherlands; 5 Department of Pathology, Leiden University Medical Center, PO Box 9600, 2300 RC Leiden, The Netherlands

## Abstract

**Introduction:**

SNPs rs2981582 and rs2981578, located in a linkage disequilibrium block (LD block) within intron 2 of the fibroblast growth factor receptor 2 gene (*FGFR2*), are associated with a mildly increased breast cancer risk. Allele-specific regulation of *FGFR2 *mRNA expression has been reported previously, but the molecular basis for the association of these variants with breast cancer has remained elusive to date.

**Methods:**

mRNA levels of *FGFR2 *and three fibroblast growth factor genes (*FGF*s) were measured in primary fibroblast and epithelial cell cultures from 98 breast cancer patients and correlated to their rs2981578 genotype. The phosphorylation levels of downstream FGFR2 targets, FGF receptor substrate 2α (FRS2α) and extracellular signal-regulated kinases 1 and 2 (ERK1/2), were quantified in skin fibroblasts exposed to FGF2. Immunohistochemical markers for angiogenesis and lymphocytic infiltrate were semiquantitatively assessed in 25 breast tumors.

**Results:**

The risk allele of rs2981578 was associated with increased *FGFR2 *mRNA levels in skin fibroblasts, but not in skin epithelial cell cultures. *FGFR2 *mRNA levels in skin fibroblasts and breast fibroblasts correlated strongly in the patients from whom both cultures were available. Tumor-derived fibroblasts expressed, on average, eight times more *FGFR2 *mRNA than the corresponding fibroblasts from normal breast tissue. Fibroblasts with higher *FGFR2 *mRNA expression showed more FRS2α and ERK1/2 phosphorylation after exposure to FGF2. In fibroblasts, higher *FGFR2 *expression correlated with higher *FGF10 *expression. In 25 breast tumors, no associations between breast tumor characteristics and fibroblast *FGFR2 *mRNA levels were found.

**Conclusions:**

The influence of rs2981578 genotypes on *FGFR2 *mRNA expression levels is cell type-dependent. Expression differences correlated well with signaling levels of the FGFR2 pathway. Our results suggest that the increased breast cancer risk associated with SNP rs2981578 is due to increased FGFR2 signaling activity in stromal fibroblasts, possibly also involving paracrine FGF10 signaling.

## Introduction

Several genome-wide association studies have shown that the minor allele of SNP rs2981582, located in intron 2 of the fibroblast growth factor receptor 2 gene (*FGFR2*), is associated with increased breast cancer risk [1-6]. The odds ratios (ORs) for this SNP are 1.23 in heterozygotes and 1.63 in homozygotes for the minor allele [1,2], but attributable risk is high because of the high frequency of the risk allele population. Fine-scale genetic mapping and resequencing of the region surrounding rs2981582 resulted in the identification of up to eight variants in a linkage disequilibrium block (LD block) within intron 2 of *FGFR2 *most strongly associated with increased breast cancer risk, including SNP rs2981578 [1,5,7]. The location of this LD block suggests that these variations somehow modify the functioning of *FGFR2*.

Meyer *et al*. [7] have shown that two SNPs within this LD block, one of them being rs2981578, alter the DNA binding affinity of octamer-binding transcription factor 1 (Oct-1), runt-related transcription factor 2 (Runx2) and CCAAT/enhancer binding protein β (C/EBPβ). Accordingly, increased expression of *FGFR2 *mRNA was observed in total RNA isolated from breast tumors of patients homozygous for the risk allele as compared to homozygotes for the major allele [7]. Paradoxically, Sun *et al*. [8] recently reported decreased expression of *FGFR2 *mRNA in normal breast tissue of homozygotes for the risk allele.

FGFR2 is one of five fibroblast growth factor (FGF) receptors known in humans to be involved in various signaling pathways that regulate processes such as cell growth, apoptosis and differentiation. Two isoforms, FGFR2-IIIb and FGFR2-IIIc, are the result of mutually exclusive alternative splicing of exon 9 or 10 of *FGFR2*. Isoform IIIb is present on epithelial cells and binds ligands FGF3, FGF7, FGF10 and FGF22 and isoform IIIc is present on mesenchymal cells and binds FGF2, FGF4, FGF6, FGF9, FGF17 and FGF18 [9]. Binding of a ligand to the receptor can activate several signaling pathways, including the mitogen-activated protein kinase (MAPK) pathway [10,11].

Downregulation of FGFR2 protein has been reported in up to 67% of breast tumors [12], whereas amplification of *FGFR2 *and upregulation of *FGFR2 *mRNA expression have been reported in less than 10% of breast tumors [10,13]. Somatic *FGFR2 *mutations are rare in breast cancer [14]. A switch from the IIIb to the IIIc isoform in tumor cells, resulting in activation of the receptor by other FGFs, has been reported in a subset of prostate cancers and in a few breast cancer cell lines [15-17].

To further unravel the mechanisms by which the SNPs in intron 2 of *FGFR2 *increase breast cancer risk, and to address the heterogeneous cellular composition of breast tumors, we studied the expression of *FGFR2 *mRNA in relation to the rs2981578 genotype in fibroblasts and epithelial cells cultured from breast tissue. We also compared the *FGFR2 *mRNA expression in fibroblasts derived from normal skin tissue, normal breast tissue and breast tumor tissue. Furthermore, we explored the functional implications of different levels of *FGFR2 *expression at the cellular level by studying the downstream MAPK pathway. Finally, we compared histological characteristics between tumors of patients with high and low *FGFR2 *levels.

## Materials and methods

### Patients

Breast tissue was obtained from 98 women who had undergone breast cancer-related surgery at the Leiden University Medical Center (LUMC) from February 2006 until December 2010 (patient demographics are given in Table 1). According to the Dutch Medical Treatment Act, anonymized samples obtained during medical treatment may be used in medical research if the patient does not object to this "secondary use" (opt-out system). Samples were coded in such a way that identifying information was not accessible by the researcher, but samples still could be linked to pathology reports by authorized clinicians. The use of residual material for this study was approved by the Department of Pathology of the LUMC (code OP 25-06). The study protocol was compliant with the Code of Conduct issued by the Dutch Federation of Medical Scientific Societies.

**Table 1:** Characteristics of the patient cohort^a^

Characteristics	Data
Total number of patients	98
*BRCA1/2 *mutation carriers, *n *(%)	10 (10%)
Diagnosis	
Mean age at diagnosis^b^, years (±SD)	55 (±14)
Invasive breast cancer, *n*	83
*In situ *breast cancer, *n*	8
Prophylactic surgery, no cancer, *n*	7
Tumor characteristics (*n *= 91)	
ER status	54 positive, 20 negative, 17 unknown

^a^SD: standard deviation; ER: estrogen receptor. ^b^Age at diagnosis of breast cancer. The seven patients who underwent prophylactic surgery were excluded from this analysis.

### Tissue culture

Fibroblasts and epithelial cells were cultured separately from skin tissue, normal breast tissue or tumor tissue by cutting the tissue patches into 2-mm × 2-mm pieces. Tissue patches were allowed to attach to the culture flask for 30 minutes, then we added DMEM/F12 medium (1:1 dilution; GIBCO/Invitrogen, Breda, the Netherlands) supplemented with 20% FCS (Bodinco, Alkmaar, The Netherlands) and antibiotics. Cells were grown at 37°C in 5% CO_2_. Each fibroblast culture was expanded in medium supplemented with 10% FCS and antibiotics. Epithelial cells were grown in HuMEC Ready Medium (GIBCO/Invitrogen) or in DermaLife K Serum-Free Keratinocyte Culture Medium (Lifeline Cell Technology, Walkersville, MD, USA) (see also Table 2). The morphology of primary cultures was in agreement with the morphology of established epithelial and mesenchymal cell lines (Figure S1 in Additional file 1).

**Table 2:** Culture medium used to culture skin epithelial cells^a^

Culture ID	Medium used
46	HuMEC
47	HuMEC
52	HuMEC
54	HuMEC
55	HuMEC
57	HuMEC
58	HuMEC
59	HuMEC
64	HuMEC
65	HuMEC
69	DermaLife
70	DermaLife
77	DermaLife
83	HuMEC
100	HuMEC
103	DermaLife
132	DermaLife
140	DermaLife
143	DermaLife
152	DermaLife
157	DermaLife
163	DermaLife
170	DermaLife
189	DermaLife
209	DermaLife

^a^Culture ID refers to the unique number assigned to a patient during the process of sample anonymization; HuMEC = HuMEC Ready Medium (GIBCO/Invitrogen, Breda, The Netherlands); DermaLife = DermaLife K Serum-Free Keratinocyte Culture Medium (Lifeline Cell Technology, Walkersville, MD, USA).

### Genotyping

Genomic DNA was isolated using the Wizard Genomic DNA Purification Kit (Promega, Leiden, The Netherlands) according to the manufacturer's instructions. Four SNPs in intron 2 of *FGFR2 *were genotyped (Table 3).

**Table 3:** Primers used to genotype four SNPs in *FGFR2*^a^

SNP	Primer	Primer sequence (5' to 3')	Product length	Annealing temperature	Further analysis
rs10736303	F	AGGGACAAATACTCCGCACA	405 bp	50°C	Sanger sequencing
	R	AGCCATCCAGCATGTTTCTC			
rs2981578	F	TGACTCTTCAAAGTTTGTTTGTTTT	295 bp	50°C	Restriction enzyme *Aci*I (New England Biolabs, Ipswich, MA, USA)
	R	GAGGAAAGGTTCCCCACACT			
rs2981582	F	AGCTCAGCTTACCCCAGACA	215 bp	58°C	Restriction enzyme *Aci*I (New England Biolabs, Ipswich, MA, USA)
	R	CGTGAGCCAAGCCTCTACTT			
rs7895676	F	CAGGTGCGGTGGCTCATGTC	345 bp	67°C	Sanger sequencing
	R	GACTTCAATGGCGGGACTCC			

^a^*FGFR2*: fibroblast growth factor receptor 2 gene; F: forward primer; R: reverse primer; bp: base pair. For each SNP, the forward and reverse primer sequences, the length of the resulting PCR product and the annealing temperature used during PCR are shown. Also shown is the technique that was used on the resulting PCR fragment to genotype the SNPs.

### Quantitative real-time PCR

Total RNA was isolated from exponentially growing fibroblasts or epithelial cells using NucleoSpin RNA II Kit (Clontech, St-Germain-en-Laye, France) according to the manufacturer's instructions. During isolation, a 30-minute DNase treatment was performed to ensure complete degradation of contaminating DNA.

A detailed description of the RT reaction and quantitative PCR (qPCR) conditions can be found in the supplementary methods section (Additional file 2). Briefly, qPCR was performed to analyze the expression of a specific gene (Table 4) using the Bio-Rad iCycler thermal cycler and the iQ SYBR Green Supermix (Bio-Rad Laboratories, Hercules, CA, USA) according to the manufacturer's recommendations. Each gene was measured at least twice in each sample. As reference genes, the TATA box binding protein (*TBP*) and heterogeneous nuclear ribonucleoprotein M (*HNRPM or HNRNPM*) (fibroblasts) genes or signal recognition particle receptor (*SRPR*), *HNRPM *and *TBP *(epithelial cells) genes were used.

**Table 4:** Primers used during real-time PCR analyses^a^

Gene and (GeneID)^b^	Sequence ID	Primer	Primer sequence (5' to 3')	Product length	Distance from poly(A) tail^c^	Exon-exon boundary
*FGF2*	NM_002006.4	F	ACCTGCAGACTGCTTTTTGCCCA	91 bp	1,731 bp	No
(2247)		R	GGTGCCACGTGAGAGCAGAGC			
*FGF7*	NM_002009.3	F	CTCAACGGCAAGTTTCCCTCCCTTTTC	80 bp	1,701 bp	No
(2252)		R	GCCTTCCAGGATTTGCTGGCCC			
*FGF10*	NM_004465.1	F	TCTTCTTCCTCCTCCTTCTCCTCTCC	148 bp	325 bp	No
(2255)		R	TCCCGCTGACCTTCCCGTTCTTCTC			
*FGFR2*^d^	NM_000141.4	F	GTCAGTGAGAACAGTAACAACAAG	192 bp	3,368 bp	Yes
(2263)		R	GTAGCCTCCAATGCGATGC			
*GADPH*	NM_002046.3	F	TTCCAGGAGCGAGATCCCT	175 bp	805 bp	Yes
(2597)		R	CACCCATGACGAACATGGG			
*HMBS*	NM_000190.3	F	CTGGTAACGGCAATGCGGCT	338 bp	1010 bp	Yes
(3145)		R	GCAGATGGCTCCGATGGTGA			
*HNRPM*	NM_005968.3	F	GAGGCCATGCTCCTGGG	85 bp	440 bp	Yes
(4670)		R	TTTAGCATCTTCCATGTGAAATCG			
*HPRT1*	NM_000194.2	F	TGACACTGGCAAAACAATGCA	94 bp	742 bp	Yes
(3251)		R	GGTCCTTTTCACCAGCAAGCT			
*SRPR*	NM_003139.2	F	CATTGCTTTTGCACGTAACCAA	70 bp	1,308 bp	Yes
(6734)		R	ATTGTCTTGCATGCGGCC			
*TBP*	NM_003194.3	F	CACGAACCACGGCACTGATT	89 bp	905 bp	Yes
(6908)		R	TTTTCTTGCTGCCAGTCTGGAC			

^a^*FGF*: fibroblast growth factor gene; *FGFR*: fibroblast growth factor receptor gene; *GAPDH*: glyceraldehyde 3-phosphate dehydrogenase gene; *HMBS*: hydroxymethylbilane synthase gene; *HNRPM*: heterogeneous nuclear ribonucleoprotein M gene; *HPRT1*: hypoxanthine phosphoribosyltransferase 1 gene; *SRPR*: signal recognition particle receptor gene; *TBP*: TATA box binding protein gene; F: forward primer; R: reverse primer; bp: base pair. For each gene, the forward and reverse primer sequences, the length of the resulting PCR product (using cDNA as template) and the distance from the poly(A) tail are shown, as is the information about whether the PCR product includes an exon-exon boundary. ^b^GeneID from EntrezGene accessed 2 June 2010. ^c^Distance from 3' end of the PCR product to 5' beginning of the poly(A) tail. ^d^Primer set for *FGFR2 *is in a region of *FGFR2 *that is present in both *FGFR2 *IIIb and *FGFR2 *IIIc.

### Fragment analysis

Isoforms FGFR2-IIIb and FGFR2-IIIc differ by the alternative splicing in of either exon 9 or exon 10 into the mature mRNA, resulting in a difference of 3 bp in length of the mature mRNA. A PCR fragment of 297 bp for *FGFR2*-IIIb or 300 bp for *FGFR2*-IIIc mRNA was amplified by PCR with 38 cycles and an annealing temperature of 70°C using the 6-carboxyfluorescein-labeled forward primer 5'-GTGGAAAAGAACGGCAGTAAATACG-3' and reverse primer 5'-CACCATACAGGCGATTAAGAAGACC-3' located in exons 8 and 11.

Fragment analysis was performed on cDNA from 14 skin epithelial cell cultures and 52 skin fibroblast cultures using the 3730xl DNA Analyzer (Applied Biosystems, Carlsbad, CA, USA) and the GeneScan 400HD ROX Size Standard (Applied Biosystems).

### Measuring FGFR2 protein levels

FGFR2 protein levels were analyzed in seven exponentially growing fibroblast cultures with high (*n *= 3) or low (*n *= 4) *FGFR2 *mRNA levels. Total protein isolation and Western blot analysis are described in detail in the supplementary methods section (Additional file 2). Each sample was measured twice on separate gels. The primary and secondary antibodies used are given in Table 5. The Odyssey Infrared Imaging System and Odyssey 3.0 software (LI-COR Biotechnology, Cambridge, UK) were used to visualize and identify the bands.

**Table 5:** Antibodies used during Western blot analyses^a^

Antigen targeted	Antibody (catalog number, manufacturer)	Clonality	Dilution	Origin
Primary antibodies				
*FGFR2*	Bek C-17 (sc-122, SZ)	Polyclonal	1 in 500	Rabbit
α-tubulin	Anti-α-tubulin (T6199, SA)	Monoclonal	1 in 8,000	Mouse
ERK1/2	p44/42 MAPK antibody (9102, CS)	Polyclonal	1 in 100	Rabbit
Phosphorylated ERK1/2	Phospho-p44/42 MAPK antibody (4377, CS)	Monoclonal	1 in 200	Rabbit
FRS2α	FRS2 H-91 (sc-8318, SZ)	Polyclonal	1 in 50	Rabbit
Phosphorylated FRS2α	Phospho-FRS2-α antibody (3864, CS)	Polyclonal	1 in 50	Rabbit
Secondary antibodies				
Mouse IgG	IRDye 680 anti-mouse IgG (926-32220, LC)	Polyclonal	1 in 7,500	Goat
Rabbit IgG	IRDye 800CW anti-rabbit IgG (926-32211, LC)	Polyclonal	1 in 5,000	Goat

^a^*FGFR2*: fibroblast growth factor receptor 2 gene; ERK1/2: extracellular signal-regulated kinases 1 and 2; FRS2α: fibroblast growth factor receptor substrate 2α; IgG: immunoglobulin G; MAPK: mitogen-activated protein kinase. Manufacturer names are abbreviated as follows: SZ: Santa Cruz Biotechnology, Santa Cruz, CA, USA; SA: Sigma-Aldrich, Zwijndrecht, The Netherlands; CS: Cell Signaling Technology, Danvers, MA, USA; LC, LI-COR Biosciences, Cambridge, UK. For each antibody, the target, manufacturer, clonality, dilution used during Western blot analysis and origin are given.

### Analyzing the phosphorylation of downstream FGFR2 targets

Five skin fibroblast cultures with high *FGFR2 *mRNA expression and five skin fibroblast cultures with low *FGFR2 *mRNA expression were randomly selected. In each experiment, one culture with high mRNA expression and one culture with low mRNA expression were compared. In each well of a six-well plate, 5 × 10^5 ^fibroblasts from the full cell culture flasks were seeded and 10% FCS medium was added. The cells were allowed to attach to the well bottom for 24 hours and were then starved in 0.5% FCS medium for 12 hours. Cells were subsequently exposed to 0 or 20 ng of FGF2 (basic fibroblast growth factor, 9952; Cell Signaling Technology, Danvers, MA, USA) in 0.5 mL of 0.5% FCS medium for 10 minutes. This length of time was chosen because in an initial experiment phosphorylation levels peaked at about 10 minutes (Figure S2 in Additional file 3).

Total protein was then isolated, and Western blot analysis was performed to assess the phosphorylation of downstream targets of FGFR2 as described in more detail in the supplementary methods section (Additional file 2). The primary and secondary antibodies that were used are given in Table 5. Phosphorylation levels after 10 minutes of exposure were normalized to phosphorylation levels before exposure.

### Determining the stroma percentage within the primary tumor

Routine H & E-stained histological tumor sections were obtained from the Department of Pathology for 50 of the 68 patients from whom skin fibroblast cultures were available. The stroma percentage within the primary tumor was estimated by two researchers as previously described [18].

### Immunohistochemistry

For the immunohistochemical analyses, patients were selected for being either homozygous for the risk allele and having high *FGFR2 *mRNA expression (*n *= 11) or for being homozygous for the major allele and having low *FGFR2 *mRNA expression (*n *= 14). Tissue sections were stained using antibodies to detect the presence of microvessels (CD31) and T lymphocytes (CD3). Microvascular density was measured by two independent observers who counted all CD31-stained blood vessels in 10 high-power fields (HPFs) randomly selected within the tumor region. The average number of blood vessels per HPF was then calculated and used in subsequent analysis. For CD3, slides were scanned using the Pannoramic MIDI digital scanner (3DHISTECH Ltd., Budapest, Hungary) and then viewed at ×10 magnification using the MIRAX Viewer 1.11 (3DHISTECH Ltd.). For each tumor, four to six representative areas of 1,800 μm × 1,200 μm were selected, and the tumor and surrounding stroma were separately analyzed using ImageJ software and the color deconvolution plug-in [19]. The area covered by the antibody was measured and normalized by dividing it by the area covered by nuclei.

### Statistical analysis

The distribution of the SNP genotypes was studied using an Excel-based Hardy-Weinberg equilibrium (HWE) calculator [20]. For all other statistical analyses, we used PASW Statistics version 17.0 software (SPSS Inc., Nieuwegein, The Netherlands).

## Results

A total of 68 skin fibroblast cultures, 44 breast fibroblast cultures, 11 tumor-derived fibroblast cultures and 25 skin epithelial cell cultures were established from 98 patients who had undergone breast cancer-related surgery at LUMC. We genotyped four SNPs in intron 2 of *FGFR2 *(Table 6). All SNPs were in HWE. SNP rs2981578 was chosen to represent all four SNPs because of its reported effect on the binding of transcription factor Runx2 and because it showed strong LD with the other three SNPs including rs2981582.

**Table 6:** Genotype distribution of four SNPs in *FGFR2*^a^

rs2981582	rs2981578	rs10736303	rs7895676	Number of patients
CT	AG	AG	CT	29
TT	GG	GG	CC	22
CC	AA	AA	TT	21
CT	GG	GG	CC	7
CC	AG	AG	CT	5
TT	AA	AA	TT	1
Total				85^b^

^a^*FGFR2*: fibroblast growth factor receptor 2 gene. The number of patients who carry each combination of the four genotypes is shown in the right-hand column. Although we have no haplotype information, the genotype distribution shows the strong linkage disequilibrium present within this region. The alleles associated with increased breast cancer risk are rs2981582: T; rs2981578: G; rs10736303: G; and rs7895676: C. ^b^For the remaining13 patients, only rs2981578 was genotyped; of these patients, 5 carried AA, 5 carried AG and 3 carried GG.

### *FGFR2 *mRNA expression in fibroblasts and epithelial cells

**Figure 1:**
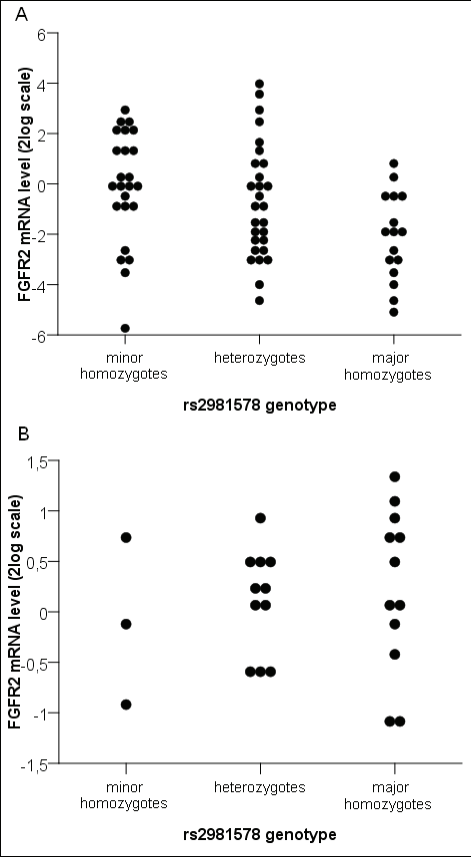
**The relationship between *FGFR2 *mRNA expression and the rs2981578 genotype in skin fibroblast and skin epithelial cell cultures**. **(A) **A significant correlation between the rs2981578 genotype and *FGFR2 *mRNA expression level as measured by quantitative real-time PCR was present in the 68 skin fibroblast cultures (*P *= 0.02; one-way ANOVA). The expression levels were log_2_-transformed and normalized to *HNRPM *and *TBP *expression. **(B) ***FGFR2 *mRNA expression and rs2981578 genotypes in 25 skin epithelial cell cultures (*P *= 0.73; one-way ANOVA). The expression levels were log_2_-transformed and normalized to *HNRPM, SRPR *and *TBP *expression. Each dot represents the expression level in one patient.

A significant correlation between the rs2981578 genotype and *FGFR2 *mRNA expression level as measured by qRT-PCR was present in the 68 skin fibroblast cultures (Figure 1A) (*P *= 0.02; one-way analysis of variance (ANOVA)). Although expression levels varied widely within each genotype group, a dosage-dependent effect was seen, with the highest average expression found among homozygotes for the minor allele (the risk allele), followed by heterozygotes, and the lowest average expression found among homozygotes for the major allele. In contrast, among 25 skin epithelial cell cultures, there were no statistically significant differences in *FGFR2 *mRNA expression levels between the three genotype groups, although the average levels of *FGFR2 *mRNA were slightly lower in carriers of one or two copies of the risk allele (Figure 1B) (*P *= 0.732; one-way ANOVA).

### *FGFR2 *mRNA expression in fibroblasts derived from different tissues

**Figure 2:**
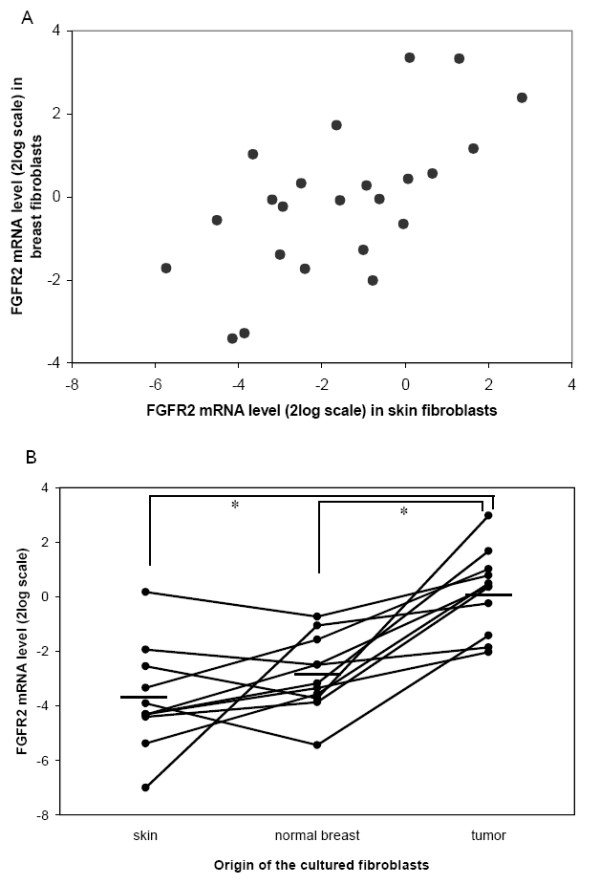
***FGFR2 *mRNA expression levels in fibroblasts cultured from different locations**. **(A) **We found a significant correlation between *FGFR2 *mRNA expression in skin fibroblasts and normal breast fibroblasts from the same patient (*n *= 22). The *FGFR2 *expression levels were log_2_-transformed and normalized to *HNRPM *and *TBP *expression. **(B) ***FGFR2 *mRNA expression in tumor-derived fibroblasts is significantly higher than in fibroblasts cultured from normal breast tissue. The *FGFR2 *expression levels were log_2_-transformed and normalized to *HNRPM *and *TBP *expression. Each dot represents *FGFR2 *expression in one patient. The lines connect the fibroblast cultures from one patient. The horizontal bars shows the mean expression for all fibroblast cultures from that origin (skin -3.8 (±1.9 SD), normal breast -2.9 (±1.4 SD), tumor tissue 0.2 (±1.5 SD)). *P < 0.001.

To address whether *FGFR2 *expression levels in skin fibroblasts correlated to those in breast fibroblasts, we cultured fibroblasts from the skin tissue as well as the normal breast tissue from 22 breast cancer patients. There was a significant correlation between the *FGFR2 *mRNA levels in the skin fibroblasts and the breast fibroblasts (Figure 2A) (Pearson's correlation coefficient = 0.64, *P *= 0.001). Expression levels were on average 2.8 times higher in the breast fibroblasts than in the skin fibroblasts (*P *= 0.001; paired *t*-test).

For 11 of these 22 patients, we also successfully grew fibroblasts from the tumor tissue. *FGFR2 *mRNA expression in tumor-derived fibroblasts was on average eight times higher than in breast fibroblasts and sixteen times higher than in skin fibroblasts (Figure 2B) (*P *= 3 × 10^-4 ^and *P *= 2 × 10^-4^, respectively; paired *t*-tests).

### *FGFR2 *mRNA isoform expression analysis in fibroblasts and epithelial cells

Since an isoform switch from FGFR2 IIIb to FGFR2 IIIc has been reported in a few breast cancer cell lines, and since this switch may contribute to tumor development, we explored whether rs2981578 genotype influences the alternative splicing of isoforms of *FGFR2*. The expected *FGFR2*-IIIb was detected in 14 epithelial cell cultures, and the expected *FGFR2*-IIIc was detected in 48 of 52 fibroblast cultures. In the remaining four fibroblast samples, *FGFR2*-IIIb was present at a level approximately 10% of that of *FGFR2*-IIIc, but the presence or absence of *FGFR2*-IIIb was not associated with rs2981578 genotype (*P *= 0.32; Pearson's χ^2 ^test).

### Correlation between *FGFR2 *and *FGF10 *mRNA expression

**Figure 3:**
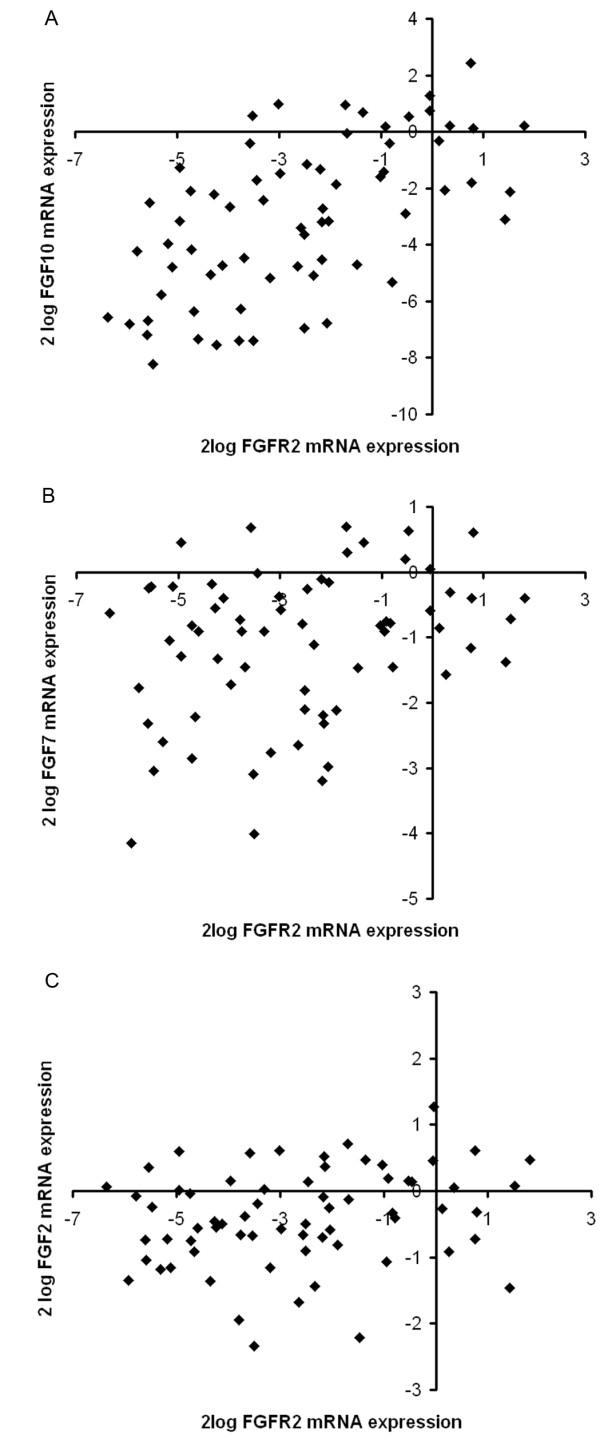
**Correlation of expression levels of *FGFR2 *and *FGF10 *mRNA in 68 skin fibroblast cultures**. No correlation was found for *FGFR2 *and *FGF2 *mRNA or *FGFR2 *and *FGF7 *mRNA levels. The data were normalized to *HNRPM *and *TBP *and log_2_-transformed. Each dot represents the expression levels from one patient. **(A) ***FGFR2 *and *FGF10 *(*P *= 8 × 10^-8^; Pearson's correlation). **(B) ***FGFR2 *and *FGF7 *(*P *= 0.02; Pearson's correlation). **(C) ***FGFR2 *and *FGF2 *(*P *= 0.07; Pearson's correlation).

To explore a possible paracrine relationship between fibroblasts and epithelial cells, the mRNA expression levels of *FGF2, FGF7 *and *FGF10 *were measured in the 68 skin fibroblast cultures and compared to their *FGFR2 *mRNA expression. *FGF2 *mRNA expression was not correlated with *FGFR2 *expression (Figure 3C) (Pearson's correlation coefficient = 0.22, *P *= 0.07). The correlation between *FGFR2 *and *FGF7 *mRNA levels in skin fibroblasts did not remain significant after correction for multiple testing (Figure 3B) (Pearson's correlation coefficient = 0.28, *P *= 0.02).

*FGFR2 *and *FGF10 *expression levels were loosely but significantly correlated in the skin fibroblast cultures (Figure 3A) (Pearson's correlation coefficient = 0.60, *P *= 8 × 10^-8^). There was also a suggestive correlation between *FGF10 *expression and rs2981578 genotype in these cultures (*P *= 0.06; one-way ANOVA) (Figure S3 in Additional file 4). In 44 fibroblast cultures from normal breast tissue, there was a suggestive correlation between *FGFR2 *and *FGF10 *mRNA levels (Spearman's ρ = 0.25, *P *= 0.11) (Figure S4 in Additional file 5).

### FGFR2 protein expression

In seven fibroblast cultures, FGFR2 protein levels were quantified by Western blot analysis and compared with the *FGFR2 *mRNA levels. The FGFR2 antibody that was used detected a full-length FGFR2 recombinant protein, but absolute protein expression levels in fibroblasts were very low, giving poor signal-to-noise ratios. Possibly as a result of this, we were unable to demonstrate a correlation between *FGFR2 *mRNA and protein levels (Spearman's ρ = -0.14, *P *= 0.76) (Figure S5 in Additional file 6).

### Phosphorylation of downstream FGFR2 targets

To investigate whether the differences in *FGFR2 *mRNA expression levels correspond with different levels of FGFR2 pathway activity, we compared the phosphorylation of downstream targets FGF receptor substrate 2α (FRS2α) and extracellular signal-regulated kinases 1 and 2 (ERK1/2) after 10 minutes of exposure to ligand FGF2 in skin fibroblast cultures with high and low *FGFR2 *mRNA levels. Duplicate measurements correlated well in all experiments (Pearson's correlation coefficient = 0.92, *P *= 8 × 10^-65^).

**Figure 4:**
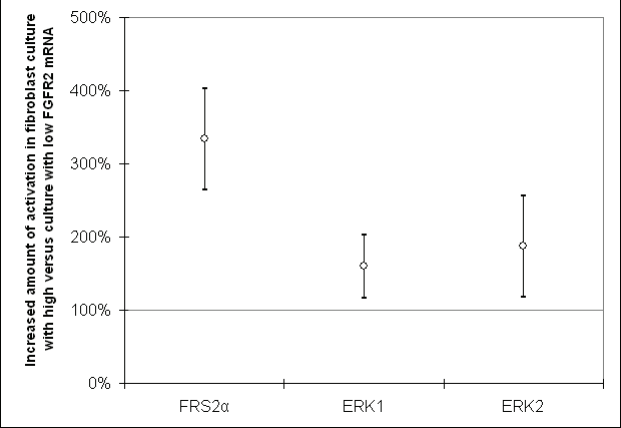
**Response of skin fibroblast cultures with high and low *FGFR2 *mRNA levels to stimulation with ligand FGF2**. Phosphorylation of downstream targets FRS2α and ERK1/2 were measured before and after 10 minutes of stimulation with FGF2. The average difference of the increase in phosphorylation is shown, which was calculated by dividing the increase in samples with high *FGFR2 *mRNA levels by the increase in samples with low *FGFR2 *mRNA levels. The 95% confidence intervals are also shown. Phosphorylation of FRS2α was measured in four fibroblast cultures: two with high and two with low *FGFR2 *mRNA levels. Phosphorylation of ERK1/2 was measured in 10 fibroblast cultures, five with high and five with low *FGFR2 *mRNA levels.

Phosphorylation of FRS2α was measured in four fibroblast cultures, two with high and two with low *FGFR2 *expression. The sample with higher *FGFR2 *expression showed a larger increase in phosphorylation of FRS2α after FGF2 was added compared to the sample with lower *FGFR2 *expression (Figure 4) (*P *= 0.18; Wilcoxon signed-rank test).

Phosphorylation of ERK1/2 increased in all skin fibroblast cultures after exposure to FGF2, and this effect was again more pronounced in the sample with higher *FGFR2 *expression in all five experiments (Figure 4) (*P *= 0.04; Wilcoxon signed-rank test).

### *FGFR2 *mRNA level and histological breast tumor characteristics

We explored tumor parameters in patients with different *FGFR2 *mRNA levels in their fibroblasts. Since the percentage of stroma in the primary tumor may vary considerably [18] and FGFR2 signaling in the stroma could influence cell proliferation, we explored whether patients with higher *FGFR2 *mRNA expression in fibroblasts had a higher stroma percentage. In the 50 patients analyzed, no correlation was present between *FGFR2 *mRNA levels in their fibroblasts and the percentage of stroma within the tumor (Spearman's ρ = 0.17, *P *= 0.23). Unfortunately, not enough tumor tissue was available from patients from whom we had obtained skin epithelial cells.

Because FGF signaling in cancer has been associated with induction of angiogenesis and lymphocytic infiltration [21], the number of microvessels was determined and T-cell infiltrate was quantified in breast tumors from patients whose fibroblasts had relatively high (*n *= 11) or relatively low (*n *= 14) *FGFR2 *expression. Interobserver agreement regarding the number of blood vessels per HPF was high (Pearson's correlation coefficient = 0.89, *P *= 1 × 10^-5^), but no correlation with *FGFR2 *mRNA levels was present (Pearson's correlation coefficient = 0.04, *P *= 0.86). No correlations between the amount of T-cell infiltrate and *FGFR2 *mRNA levels in the fibroblasts were detected either (T cells in stroma: Pearson's correlation coefficient = 0.04, *P *= 0.86; T cells in tumor: Pearson's correlation coefficient = 0.15, *P *= 0.52).

Finally, since the SNPs in intron 2 of *FGFR2 *are mainly associated with estrogen receptor-positive (ER-positive) breast tumors [1,22], we studied a possible correlation between *FGFR2 *mRNA levels and ER status of the tumor in 50 patients, but could detect none (*P *= 0.60; logistic regression).

## Discussion

Aberrant FGF signaling has been implicated in the pathogenesis of multiple types of cancer, including breast cancer [11,23]. Germline genetic variation in an LD region within intron 2 of *FGFR2 *has been associated with a modestly increased breast cancer risk. Fine-scale genetic mapping and functional analyses identified up to eight SNPs, including SNP rs2981578, as likely causal variants [1,5,7]. It was demonstrated that these SNPs influence mRNA expression levels of the *FGFR2 *gene. Despite this progress, substantial uncertainty remains regarding how subtle modulation of *FGFR2 *expression levels may modify breast cancer risk. In addition, opposite effects of intron 2 SNP genotypes on *FGFR2 *mRNA expression have been reported in tumor tissue and normal breast tissue [7,8].

We found, on average, higher *FGFR2 *mRNA levels in skin fibroblast cultures of heterozygotes and homozygotes for the risk allele of SNP rs2981578 relative to homozygotes for the major allele. This is in agreement with measurements of *FGFR2 *mRNA expression in total RNA from breast tumor tissue [7]. However, no effect of genotype was observed in our skin epithelial cell cultures. Also, epithelial breast tumor cells express lower levels of FGFR2 than surrounding normal breast epithelium [12]. Soluble factors secreted or recruited by tumor cells may induce expression changes in neighboring normal cells, leading to gene expression differences between tumor-derived stromal fibroblasts and normal breast stroma [24]. In the 11 patients from whom we obtained fibroblasts from skin, normal breast tissue and breast tumor tissue, *FGFR2 *expression levels in the tumor-derived fibroblasts were consistently higher than in the fibroblasts from normal breast tissue. A similar finding was made recently in cell cultures from patients with esophageal cancer [25]. Unfortunately, our sample set was too small to conclusively establish whether expression levels of *FGFR2 *mRNA in tumor-derived fibroblasts are similarly associated with SNP genotypes in intron 2 as they are in skin-derived fibroblasts. Taken together, however, these data suggest that the association between genotype and *FGFR2 *expression observed by Meyer *et al*. [7] in total breast tumor homogenates was derived from cancer-associated fibroblasts rather than from the tumor (or epithelial) component. Because the transcription factors Oct-1/Runx2 and C/EBPβ display differential binding to these SNP alleles in epithelial breast tumor cells, it will be interesting to study this in other cell types constituting breast tumor stroma.

Our results in fibroblast and epithelial cell cultures are at odds with those obtained by Sun *et al*. [8], who reported lower *FGFR2 *mRNA expression in normal breast tissue from homozygotes for the risk allele. However, the differences reported by Sun *et al*. were small and the correlation was weak. In our epithelial cell cultures, there was a trend toward lower *FGFR2 *mRNA expression in carriers of one or two copies of the risk allele, but the differences were very small. The insufficient statistical power of our studies or the differences in experimental design (whole tissue analysis versus analysis of cultured epithelial cells) may underlie these different outcomes.

The variation in *FGFR2 *mRNA expression between individuals with the same intron 2 genotype is wide in both fibroblasts and epithelial cells. Apparently, the causal variants in intron 2 of *FGFR2 *only partly determine the *FGFR2 *mRNA expression level. We have shown that in skin fibroblasts, higher *FGFR2 *mRNA levels correspond to higher activity of the FGFR2 pathway upon stimulation by ligand FGF2, indicating that the allelic status of the intron 2 SNPs is functional at the FGF signaling level. Cells with higher *FGFR2 *expression probably respond differently to the same fibroblast growth factor concentration in their microenvironment [26]. Thus, the overall activity of the FGFR2 pathway might be a more accurate predictor of breast cancer risk than rs2981578 or rs2981582 genotype status, although it is presently unclear through which cell type and in which developmental phase of the mammary gland this activity contributes most to this risk.

Fibroblasts have a well-recognized role in the carcinogenic process as remodelers of the extracellular matrix in tumor stroma and as a source of paracrine growth factors that influence the growth of carcinoma cells [27,28]. Unequivocal evidence that paracrine FGF released from breast tumor stroma functions to promote tumorigenesis is lacking, but, intriguingly, we found a strong correlation between *FGFR2 *and *FGF10 *mRNA expression levels in the cultured skin fibroblasts. We have not investigated the mechanism underlying this correlation, nor have we been able to establish that it also holds true for tumor-derived fibroblasts. Since FGF10 is known to be secreted by fibroblasts and to bind specifically to the FGFR2-IIIb isoform expressed on epithelial cells, however, our findings would fit a model of paracrine tumor-stroma interaction. The FGFR2-IIIb-FGF10 interaction plays a key role in the normal embryological and postnatal development of the mammary glands in mice [29,30]. Mice deficient in *Fgf10 *or *Fgfr2b *fail to develop normal mammary glands. Apparently, Fgfr2b signaling is crucial for the survival and proliferation of the mammary luminal epithelial cells but does not affect the regenerative potential of the mammary epithelial progenitor cells. *Fgf10 *overexpression in the stromal compartment of the murine prostate results in epithelial cell hyperproliferation [31]. Also, in humans, FGF10 is thought to stimulate epithelial cell proliferation [32].

Thus, it is conceivable that, in the human breast, slightly increased levels of FGFR2 and FGF10 result in a slightly increased ductal branching. If this primarily involved the luminal epithelial component of the breast tissue as it does in mice, this would explain why the increased breast cancer risk conferred by *FGFR2 *intron 2 SNPs is mostly restricted to ER-positive tumors [22,33]. High FGF2 and/or FGFR2 protein levels have been correlated with high ER levels in breast cancer [34,35]. Our cohort was probably underpowered to demonstrate a correlation between ER status of the tumor and *FGFR2 *mRNA expression levels in skin fibroblasts. FGF7, also secreted by breast fibroblasts, has similarly been suggested to act as a paracrine growth factor in human breast cancer [36], but our data do not demonstrate a significant link between *FGF7 *and *FGFR2 *expression.

In pancreatic cancer, FGF10 is found in stromal cells, close to the tumor cells, and is thought to interact with FGFR2-IIIb on the tumor cells, thereby inducing cell migration and invasion [37]. Since the downstream effects of activation of FGFR2 by different FGFs in different cell types are very diverse and could include apoptosis, cell proliferation and angiogenesis [11], it is difficult to predict which of these effects could mechanistically explain the increased breast cancer risk associated with intron 2 SNPs. Here we have explored some of the end points of these processes in breast tumors from patients with different *FGFR2 *mRNA levels in their fibroblasts. Tsunoda *et al*. [38] reported increased T-lymphocyte and macrophage infiltration into a newly inoculated tumor when they injected FGF2 into this tumor. In mice, overexpression of *Fgf10 *leads to highly vascularized tumors in immunocompetent mice [39]. However, we found no correlation between *FGFR2 *mRNA levels and, respectively, stroma percentage of the tumor, microvessel density and T-cell infiltrate. Our sample size may have been too small to detect any existing differences, in particular because these differences can be expected to be small, given the mildly increased breast cancer risks conferred by *FGFR2 *SNPs.

A limitation of our study is that our observations were based on cultured cells from surgically removed tissues. Culture conditions between fibroblasts and epithelial cells were necessarily different and may have influenced some of our findings, but this enabled us to study RNA expression levels by cell type instead of by heterogeneous tissue. It has long been known that the phenotype of fibroblasts can differ depending on anatomical site [40-43]. Indeed, we did observe absolute differences in *FGFR2 *mRNA expression levels between fibroblasts from skin tissue and those from normal breast tissue, but we demonstrated that these correlate very well (Figure 2).

## Conclusions

In conclusion, it is likely that the causative variants tagged by rs2981578 in intron 2 of the *FGFR2 *gene cause a higher breast cancer risk by influencing *FGFR2 *expression levels. Here we have shown that the effect of intron 2 SNPs on *FGFR2 *expression is cell type-specific. Different effects were observed in skin fibroblasts and epithelial cells. Tissue specificity for expression quantitative trait loci is common [44] and clearly also applies to *FGFR2*.

In addition, we observed differences in the levels of *FGFR2 *mRNA expression between fibroblasts derived from normal breast tissue or from tumor tissue, which strongly supports a holistic model to explain FGFR2-related breast cancer risk [45] rather than one assuming cell autonomous effects of *FGFR2 *expression modulation. Our finding that individuals in whom intrinsically higher levels of *FGFR2 *are expressed in their skin fibroblasts also have higher levels of *FGF10 *suggests that the increased breast cancer risk might be due to a stronger paracrine effect between stromal and tumor cells involving FGF10 signaling. The fact that the association between *FGFR2 *and *FGF10 *expression was not observed by Meyer *et al*. [7] in 45 normal breast tissue samples underscores the importance of analyzing the various constituting cell types separately in sufficiently large cohorts. Since we limited our search for such associations to FGFs secreted by fibroblasts, and given that the *FGFR2 *and *FGF10 *genes are located on different chromosomes, it will be important to extend these analyses to genome-wide expression differences between different cell types in various *FGFR2 *genotype backgrounds.

## Abbreviations

bp: base pairs; C/EBPβ: CCAAT/enhancer binding protein β; CI: confidence interval; ER: estrogen receptor; ERK1/2: extracellular signal-regulated kinases 1 and 2; FCS: fetal calf serum; *FGFR2*: fibroblast growth factor receptor 2 gene; FGF: fibroblast growth factor; FRS2α: fibroblast growth factor receptor substrate 2α; H & E: hematoxylin and eosin; HNRPM: heterogeneous nuclear ribonucleoprotein M; HPF: high-power field; HWE: Hardy-Weinberg equilibrium; LD: linkage disequilibrium; MAPK: mitogen-activated protein kinase; Oct-1: octamer-binding transcription factor 1; OR: odds ratio; PCR: polymerase chain reaction; qPCR: quantitative real-time PCR; Runx2: runt-related transcription factor 2; SNP: single-nucleotide polymorphism; SRPR: signal recognition particle receptor; TBP: TATA box binding protein.

## Competing interests

The authors declare that they have no competing interests.

## Authors' contributions

The experiments were performed by PH, MD, MG and AM. The patient material was provided by RT and VS. The cells were cultured by FB and MV. The stroma percentage of 50 tumors was analyzed by EK and WM. The experiments were designed, analyzed and interpreted by PH, CA and PD. EZ helped with the statistical analyses and the interpretation of the data. The manuscript was drafted by PH and critically revised by MV, CA and PD. All authors read and approved the final manuscript.

## Supplementary Material

Additional file 1 **Figure S1**. **(A) **Typical fibroblast and **(B) **typical epithelial cell cultures (original magnification, ×10). Click here for file

Additional file 2 **Supplementary methods**. This file contains supplementary methods regarding the quantitative real-time PCR and Western blot analyses. Click here for file

Additional file 3 **Figure S2**. Phosphorylation levels of downstream targets of FGFR2 after different periods of stimulation with FGF2 in one fibroblast sample (sample from experiment 1 with high *FGFR2 *mRNA levels). **(A) **Using one of the blots as an example, we show phosphorylated ERK1/2 examined after 0, 1, 5, 10 and 20 minutes. H: lanes with total protein from a fibroblast sample with high *FGFR2 *mRNA level; L: lanes from a fibroblast sample with low *FGFR2 *mRNA level; M: lane with the length marker; T: tubulin, E1/2 ERK1/2. **(B) **Phosphorylation levels of FRS2α, ERK1 and ERK2 are shown as fold increases compared to the phosphorylation levels at 0 minutes of stimulation. Click here for file

Additional file 4 **Figure S3**. The relationship between *FGF10 *mRNA expression and the rs2981578 genotype in 68 skin fibroblast cultures (*P *= 0.06; one-way ANOVA). The expression levels were log_2_-transformed and normalized to *HNRPM *and *TBP *expression. Click here for file

Additional file 5 **Figure S4**. Correlation of expression levels of *FGFR2 *and *FGF10 *mRNA in 44 breast fibroblast cultures. *FGFR2 *and *FGF10 *expression levels were normalized to *HNRPM *and *TBP *and log_2_-transformed. Each dot represents the expression levels of one patient (Spearman's ρ = 0.25, *P *= 0.11). Click here for file

Additional file 6 **Figure S5**. Western blot analysis measuring FGFR2 protein levels in fibroblasts. **(A) **Western blot showing results for seven different fibroblast cultures. H: lanes with total protein from fibroblasts with high *FGFR2 *mRNA levels; L: lanes with total protein from fibroblasts with low *FGFR2 *mRNA levels; empty: empty lane; M: lane with length marker; recomb: lane with pure recombinant FGFR2. **(B) ***FGFR2 *mRNA levels and FGFR2 protein levels in the seven fibroblast samples. The *FGFR2 *mRNA results were normalized to *HNRPM *and *TBP *and log_2_-transformed. The FGFR2 protein results were normalized to α-tubulin. Each dot represents the results for one fibroblast sample. Click here for file

## References

[B1] Easton DF Pooley KA Dunning AM Pharoah PD Thompson D Ballinger DG Struewing JP Morrison J Field H Luben R Wareham N Ahmed S Healey CS Bowman R SEARCH collaborators Meyer KB Haiman CA Kolonel LK Henderson BE Le Marchand L Brennan P Sangrajrang S Gaborieau V Odefrey F Shen CY Wu PE Wang HC Eccles D Evans DG Genome-wide association study identifies novel breast cancer susceptibility loci Nature 2007 447 1087 1093 10.1038/nature05887 17529967 PMC2714974

[B2] Hunter DJ Kraft P Jacobs KB Cox DG Yeager M Hankinson SE Wacholder S Wang Z Welch R Hutchinson A Wang J Yu K Chatterjee N Orr N Willett WC Colditz GA Ziegler RG Berg CD Buys SS McCarty CA Feigelson HS Calle EE Thun MJ Hayes RB Tucker M Gerhard DS Fraumeni JF Jr Hoover RN Thomas G Chanock SJ A genome-wide association study identifies alleles in *FGFR2 *associated with risk of sporadic postmenopausal breast cancer Nat Genet 2007 39 870 874 10.1038/ng2075 17529973 PMC3493132

[B3] Kawase T Matsuo K Suzuki T Hiraki A Watanabe M Iwata H Tanaka H Tajima K *FGFR2 *intronic polymorphisms interact with reproductive risk factors of breast cancer: results of a case control study in Japan Int J Cancer 2009 125 1946 1952 10.1002/ijc.24505 19582883

[B4] Liang J Chen P Hu Z Zhou X Chen L Li M Wang Y Tang J Wang H Shen H Genetic variants in fibroblast growth factor receptor 2 (FGFR2) contribute to susceptibility of breast cancer in Chinese women Carcinogenesis 2008 29 2341 2346 10.1093/carcin/bgn235 18845558

[B5] Udler MS Meyer KB Pooley KA Karlins E Struewing JP Zhang J Doody DR MacArthur S Tyrer J Pharoah PD Luben R Bernstein L Kolonel LN Henderson BE Le Marchand L Ursin G Press MF Brennan P Sangrajrang S Gaborieau V Odefrey F Shen CY Wu PE Wang HC Kang D Yoo KY Noh DY Ahn SH Ponder BA Haiman CA SEARCH Collaborators *FGFR2 *variants and breast cancer risk: fine-scale mapping using African American studies and analysis of chromatin conformation Hum Mol Genet 2009 18 1692 1703 10.1093/hmg/ddp078 19223389 PMC2733817

[B6] Zheng W Cai Q Signorello LB Long J Hargreaves MK Deming SL Li G Li C Cui Y Blot WJ Evaluation of 11 breast cancer susceptibility loci in African-American women Cancer Epidemiol Biomarkers Prev 2009 18 2761 2764 10.1158/1055-9965.EPI-09-0624 19789366 PMC2759857

[B7] Meyer KB Maia AT O'Reilly M Teschendorff AE Chin SF Caldas C Ponder BA Allele-specific up-regulation of *FGFR2 *increases susceptibility to breast cancer PLoS Biol 2008 6 e108 10.1371/journal.pbio.0060108 18462018 PMC2365982

[B8] Sun C Olopade OI Di RA rs2981582 is associated with *FGFR2 *expression in normal breast Cancer Genet Cytogenet 2010 197 193 194 10.1016/j.cancergencyto.2009.11.006 20193855 PMC2831800

[B9] Eswarakumar VP Lax I Schlessinger J Cellular signaling by fibroblast growth factor receptors Cytokine Growth Factor Rev 2005 16 139 149 10.1016/j.cytogfr.2005.01.001 15863030

[B10] Knights V Cook SJ De-regulated FGF receptors as therapeutic targets in cancer Pharmacol Ther 2010 125 105 117 10.1016/j.pharmthera.2009.10.001 19874848

[B11] Turner N Grose R Fibroblast growth factor signalling: from development to cancer Nat Rev Cancer 2010 10 116 129 10.1038/nrc2780 20094046

[B12] Zhu X Asa SL Ezzat S Genetic and epigenetic mechanisms down-regulate FGF receptor 2 to induce melanoma-associated antigen A in breast cancer Am J Pathol 2010 176 2333 2343 10.2353/ajpath.2010.091049 20348248 PMC2861098

[B13] Penault-Llorca F Bertucci F Adélaïde J Parc P Coulier F Jacquemier J Birnbaum D deLapeyrière O Expression of *FGF *and *FGF *receptor genes in human breast cancer Int J Cancer 1995 61 170 176 10.1002/ijc.2910610205 7705943

[B14] Stephens P Edkins S Davies H Greenman C Cox C Hunter C Bignell G Teague J Smith R Stevens C O'Meara S Parker A Tarpey P Avis T Barthorpe A Brackenbury L Buck G Butler A Clements J Cole J Dicks E Edwards K Forbes S Gorton M Gray K Halliday K Harrison R Hills K Hinton J Jones D A screen of the complete protein kinase gene family identifies diverse patterns of somatic mutations in human breast cancer Nat Genet 2005 37 590 592 10.1038/ng1571 15908952

[B15] Kwabi-Addo B Ropiquet F Giri D Ittmann M Alternative splicing of fibroblast growth factor receptors in human prostate cancer Prostate 2001 46 163 172 10.1002/1097-0045(20010201)46:2<163::AID-PROS1020>3.0.CO;2-T 11170144

[B16] Luqmani YA Bansal GS Mortimer C Buluwela L Coombes RC Expression of FGFR2 BEK and K-SAM mRNA variants in normal and malignant human breast Eur J Cancer 1996 32A 518 524 8814701 10.1016/0959-8049(95)00563-3

[B17] Zhu X Asa SL Ezzat S Histone-acetylated control of fibroblast growth factor receptor 2 intron 2 polymorphisms and isoform splicing in breast cancer Mol Endocrinol 2009 23 1397 1405 10.1210/me.2009-0071 19497954 PMC2737561

[B18] de Kruijf EM van Nes JG van de Velde CJ Putter H Smit VT Liefers GJ Kuppen PJ Tollenaar RA Mesker WE Tumor-stroma ratio in the primary tumor is a prognostic factor in early breast cancer patients, especially in triple-negative carcinoma patients Breast Cancer Res Treat 2011 125 687 696 10.1007/s10549-010-0855-6 20361254

[B19] Ruifrok AC Johnston DA Quantification of histochemical staining by color deconvolution Anal Quant Cytol Histol 2001 23 291 299 11531144

[B20] Court lab HW calculator http://www.tufts.edu/~mcourt01/Documents/Court%20lab%20-%20HW%20calculator.xls

[B21] Acevedo VD Ittmann M Spencer DM Paths of FGFR-driven tumorigenesis Cell Cycle 2009 8 580 588 10.4161/cc.8.4.7657 19182515 PMC7441526

[B22] Garcia-Closas M Hall P Nevanlinna H Pooley K Morrison J Richesson DA Bojesen SE Nordestgaard BG Axelsson CK Arias JI Milne RL Ribas G González-Neira A Benítez J Zamora P Brauch H Justenhoven C Hamann U Ko YD Bruening T Haas S Dörk T Schürmann P Hillemanns P Bogdanova N Bremer M Karstens JH Fagerholm R Aaltonen K Aittomäki K Heterogeneity of breast cancer associations with five susceptibility loci by clinical and pathological characteristics PLoS Genet 2008 4 e1000054 10.1371/journal.pgen.1000054 18437204 PMC2291027

[B23] Katoh M Cancer genomics and genetics of FGFR2 Int J Oncol 2008 33 233 237 18636142

[B24] Singer CF Gschwantler-Kaulich D Fink-Retter A Haas C Hudelist G Czerwenka K Kubista E Differential gene expression profile in breast cancer-derived stromal fibroblasts Breast Cancer Res Treat 2008 110 273 281 10.1007/s10549-007-9725-2 17899370

[B25] Zhang C Fu L Fu J Hu L Yang H Rong TH Li Y Liu H Fu SB Zeng YX Guan XY Fibroblast growth factor receptor 2-positive fibroblasts provide a suitable microenvironment for tumor development and progression in esophageal carcinoma Clin Cancer Res 2009 15 4017 4027 10.1158/1078-0432.CCR-08-2824 19509166

[B26] Garcia-Maya M Anderson AA Kendal CE Kenny AV Edwards-Ingram LC Holladay A Saffell JL Ligand concentration is a driver of divergent signaling and pleiotropic cellular responses to FGF J Cell Physiol 2006 206 386 393 10.1002/jcp.20483 16155940

[B27] Bhowmick NA Neilson EG Moses HL Stromal fibroblasts in cancer initiation and progression Nature 2004 432 332 337 10.1038/nature03096 15549095 PMC3050735

[B28] Kalluri R Zeisberg M Fibroblasts in cancer Nat Rev Cancer 2006 6 392 401 10.1038/nrc1877 16572188

[B29] Mailleux AA Spencer-Dene B Dillon C Ndiaye D Savona-Baron C Itoh N Kato S Dickson C Thiery JP Bellusci S Role of FGF10/FGFR2b signaling during mammary gland development in the mouse embryo Development 2002 129 53 60 11782400 10.1242/dev.129.1.53

[B30] Parsa S Ramasamy SK De Langhe S Gupte VV Haigh JJ Medina D Bellusci S Terminal end bud maintenance in mammary gland is dependent upon FGFR2b signaling Dev Biol 2008 317 121 131 10.1016/j.ydbio.2008.02.014 18381212

[B31] Abate-Shen C Shen MM FGF signaling in prostate tumorigenesis: new insights into epithelial-stromal interactions Cancer Cell 2007 12 495 497 10.1016/j.ccr.2007.11.021 18068626

[B32] Katoh M Katoh M FGFR2 and WDR11 are neighboring oncogene and tumor suppressor gene on human chromosome 10q26 Int J Oncol 2003 22 1155 1159 12684685

[B33] Stacey SN Manolescu A Sulem P Thorlacius S Gudjonsson SA Jonsson GF Jakobsdottir M Bergthorsson JT Gudmundsson J Aben KK Strobbe LJ Swinkels DW van Engelenburg KC Henderson BE Kolonel LN Le Marchand L Millastre E Andres R Saez B Lambea J Godino J Polo E Tres A Picelli S Rantala J Margolin S Jonsson T Sigurdsson H Jonsdottir T Hrafnkelsson J Common variants on chromosome 5p12 confer susceptibility to estrogen receptor-positive breast cancer Nat Genet 2008 40 703 706 10.1038/ng.131 18438407

[B34] Smith K Fox SB Whitehouse R Taylor M Greenall M Clarke J Harris AL Upregulation of basic fibroblast growth factor in breast carcinoma and its relationship to vascular density, oestrogen receptor, epidermal growth factor receptor and survival Ann Oncol 1999 10 707 713 10.1023/A:1008303614441 10442194

[B35] Tozlu S Girault I Vacher S Vendrell J Andrieu C Spyratos F Cohen P Lidereau R Bieche I Identification of novel genes that co-cluster with estrogen receptor α in breast tumor biopsy specimens, using a large-scale real-time reverse transcription-PCR approach Endocr Relat Cancer 2006 13 1109 1120 10.1677/erc.1.01120 17158757

[B36] Hishikawa Y Tamaru N Ejima K Hayashi T Koji T Expression of keratinocyte growth factor and its receptor in human breast cancer: its inhibitory role in the induction of apoptosis possibly through the overexpression of Bcl-2 Arch Histol Cytol 2004 67 455 464 10.1679/aohc.67.455 15781986

[B37] Nomura S Yoshitomi H Takano S Shida T Kobayashi S Ohtsuka M Kimura F Shimizu H Yoshidome H Kato A Miyazaki M FGF10/FGFR2 signal induces cell migration and invasion in pancreatic cancer Br J Cancer 2008 99 305 313 10.1038/sj.bjc.6604473 18594526 PMC2480967

[B38] Tsunoda S Sakurai H Saito Y Ueno Y Koizumi K Saiki I Massive T-lymphocyte infiltration into the host stroma is essential for fibroblast growth factor-2-promoted growth and metastasis of mammary tumors via neovascular stability Am J Pathol 2009 174 671 683 10.2353/ajpath.2009.080471 19116363 PMC2630574

[B39] Theodorou V Boer M Weigelt B Jonkers J van der Valk M Hilkens J *Fgf10 *is an oncogene activated by MMTV insertional mutagenesis in mouse mammary tumors and overexpressed in a subset of human breast carcinomas Oncogene 2004 23 6047 6055 10.1038/sj.onc.1207816 15208658

[B40] Sorrell JM Caplan AI Fibroblasts: a diverse population at the center of it all Int Rev Cell Mol Biol 2009 276 161 214 19584013 10.1016/S1937-6448(09)76004-6

[B41] Chang HY Chi JT Dudoit S Bondre C van de Rijn M Botstein D Brown PO Diversity, topographic differentiation, and positional memory in human fibroblasts Proc Natl Acad Sci USA 2002 99 12877 12882 10.1073/pnas.162488599 12297622 PMC130553

[B42] Rinn JL Bondre C Gladstone HB Brown PO Chang HY Anatomic demarcation by positional variation in fibroblast gene expression programs PLoS Genet 2006 2 e119 10.1371/journal.pgen.0020119 16895450 PMC1523235

[B43] Nolte SV Xu W Rennekampff HO Rodemann HP Diversity of fibroblasts: a review on implications for skin tissue engineering Cells Tissues Organs 2008 187 165 176 10.1159/000111805 18042973

[B44] Huang GJ Shifman S Valdar W Johannesson M Yalcin B Taylor MS Taylor JM Mott R Flint J High resolution mapping of expression QTLs in heterogeneous stock mice in multiple tissues Genome Res 2009 19 1133 1140 10.1101/gr.088120.108 19376938 PMC2694476

[B45] Hanahan D Weinberg RA The hallmarks of cancer Cell 2000 100 57 70 10.1016/S0092-8674(00)81683-9 10647931

