# Supplementary figures and images for: Allele-specific regulation of FGFR2 expression is cell type-dependent and may increase breast cancer risk through a paracrine stimulus involving FGF10
Source: Breast Cancer Res. 2011 Jul 18;13(4):R72. doi: 10.1186/bcr2917 (PMC3236336; doi:10.1186/bcr2917)

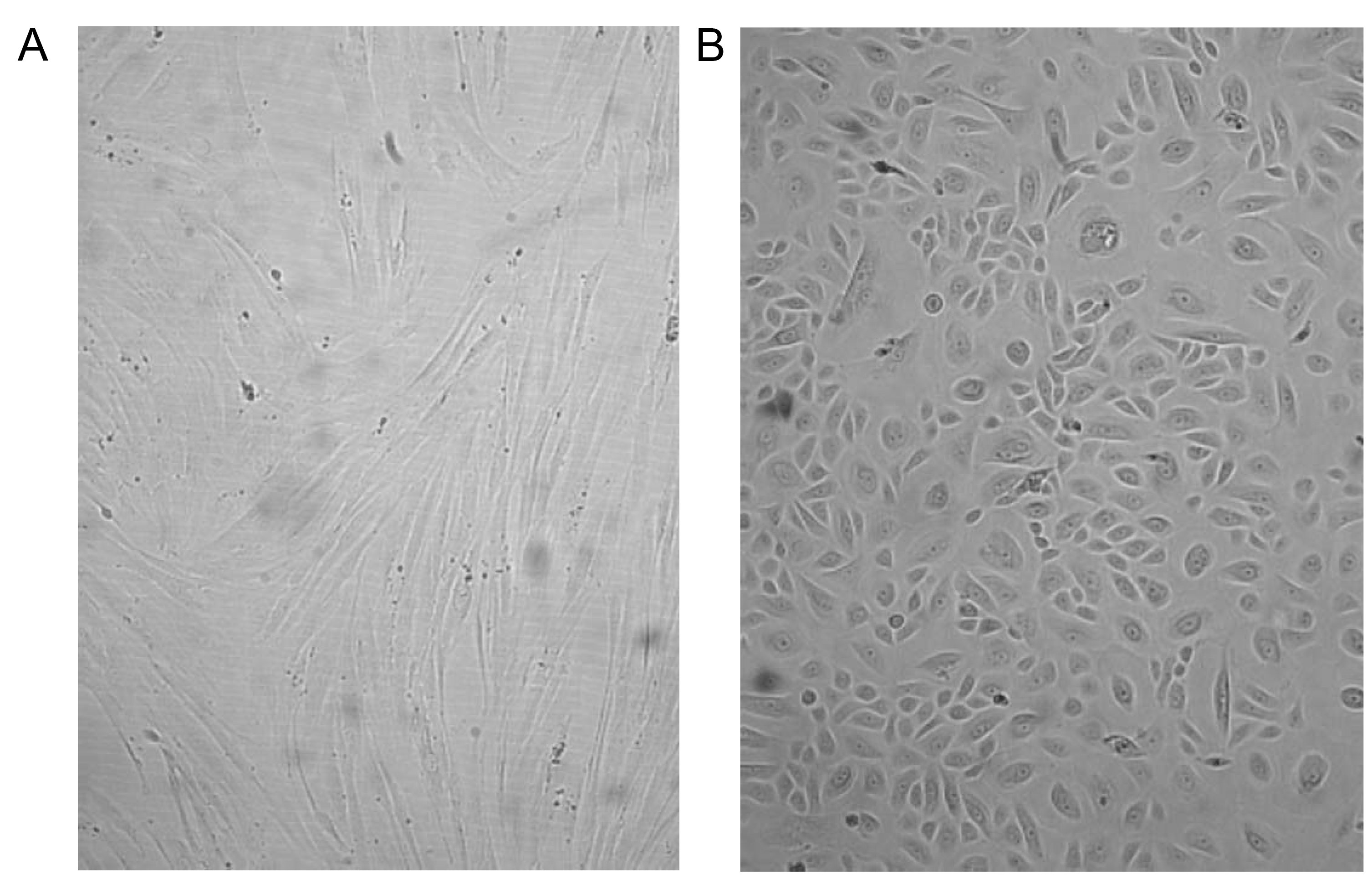

Supplement: Additional file 1 — Figure S1. (A) Typical fibroblast and (B) typical epithelial cell cultures (original magnification, ×10). [file bcr2917-S1.TIFF]

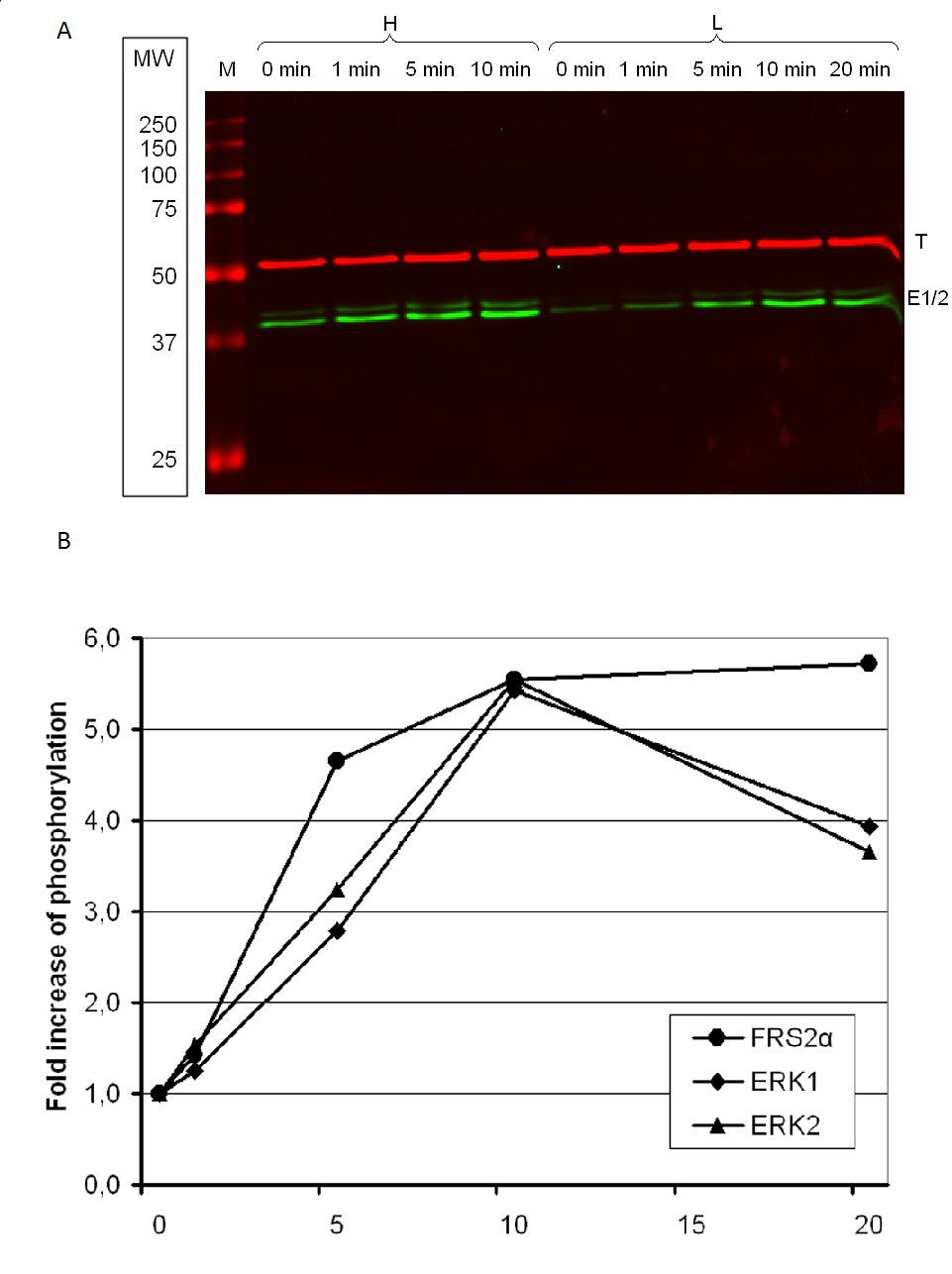

Supplement: Additional file 3 — Figure S2. Phosphorylation levels of downstream targets of FGFR2 after different periods of stimulation with FGF2 in one fibroblast sample (sample from experiment 1 with high FGFR2 mRNA levels). (A) Using one of the blots as an example, we show phosphorylated ERK1/2 examined after 0, 1, 5, 10 and 20 minutes. H: lanes with total protein from a fibroblast sample with high FGFR2 mRNA level; L: lanes from a fibroblast sample with low FGFR2 mRNA level; M: lane with the length marker; T: tubulin, E1/2 ERK1/2. (B) Phosphorylation levels of FRS2α, ERK1 and ERK2 are shown as fold increases compared to the phosphorylation levels at 0 minutes of stimulation. [file bcr2917-S3.TIFF]

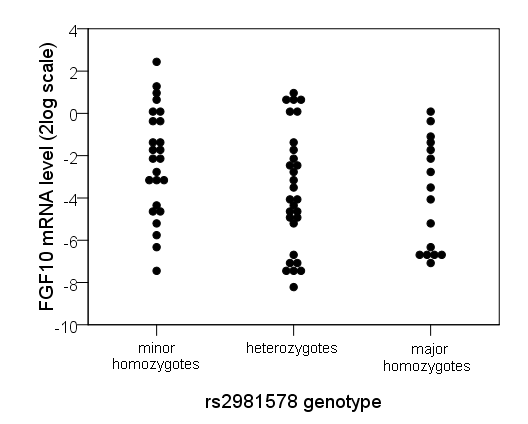

Supplement: Additional file 4 — Figure S3. The relationship between FGF10 mRNA expression and the rs2981578 genotype in 68 skin fibroblast cultures (P = 0.06; one-way ANOVA). The expression levels were log2-transformed and normalized to HNRPM and TBP expression. [file bcr2917-S4.TIFF]

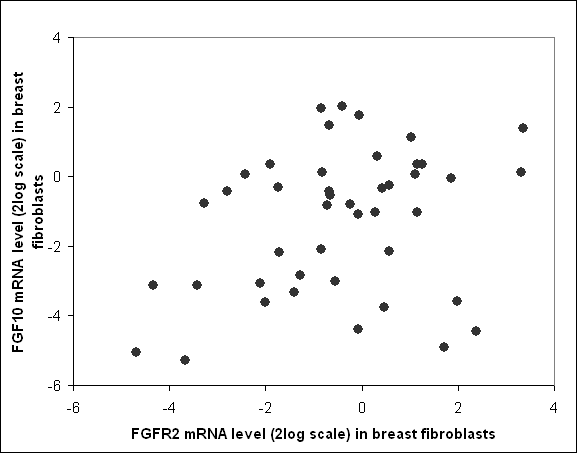

Supplement: Additional file 5 — Figure S4. Correlation of expression levels of FGFR2 and FGF10 mRNA in 44 breast fibroblast cultures. FGFR2 and FGF10 expression levels were normalized to HNRPM and TBP and log2-transformed. Each dot represents the expression levels of one patient (Spearman's ρ = 0.25, P = 0.11). [file bcr2917-S5.TIFF]

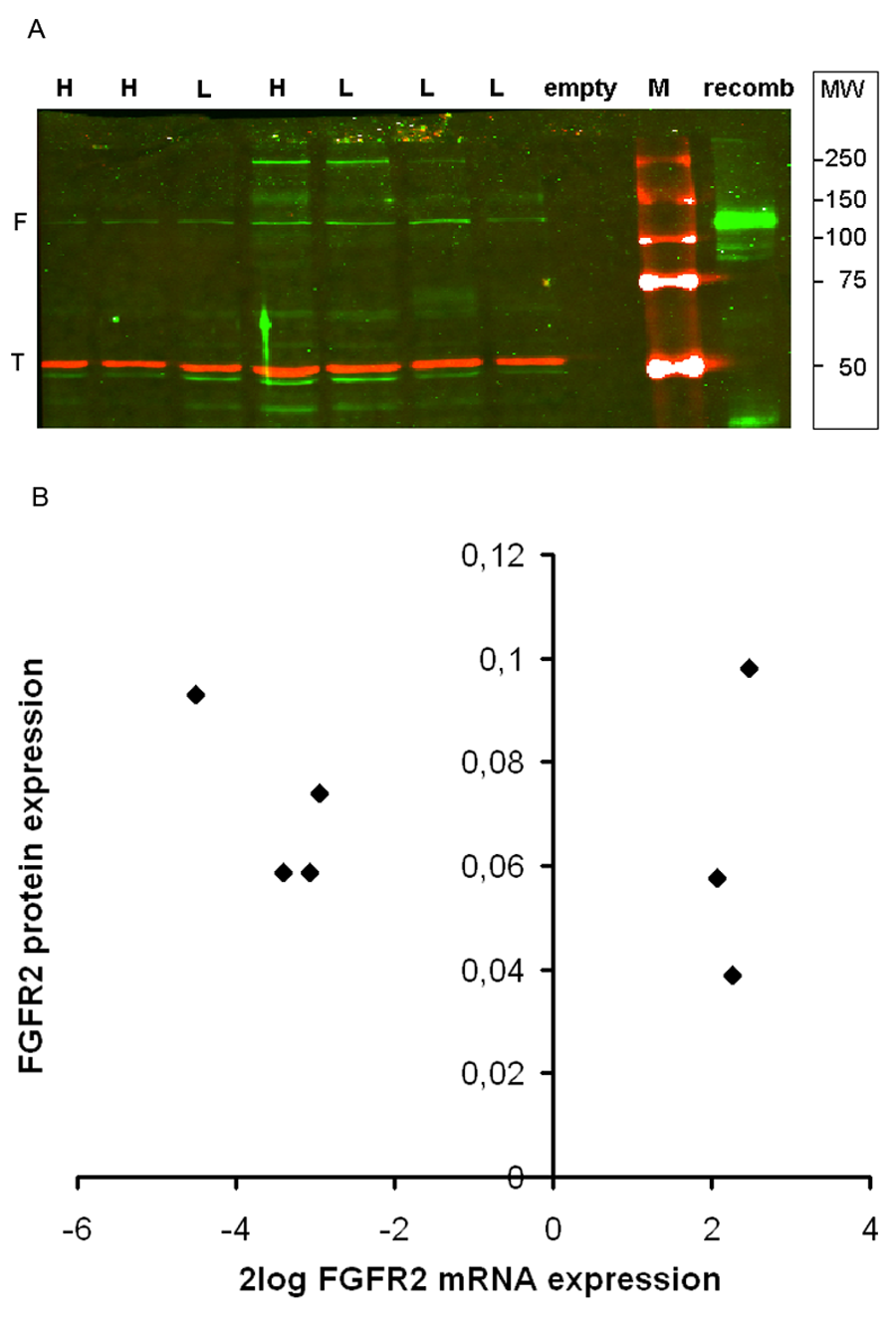

Supplement: Additional file 6 — Figure S5. Western blot analysis measuring FGFR2 protein levels in fibroblasts. (A) Western blot showing results for seven different fibroblast cultures. H: lanes with total protein from fibroblasts with high FGFR2 mRNA levels; L: lanes with total protein from fibroblasts with low FGFR2 mRNA levels; empty: empty lane; M: lane with length marker; recomb: lane with pure recombinant FGFR2. (B) FGFR2 mRNA levels and FGFR2 protein levels in the seven fibroblast samples. The FGFR2 mRNA results were normalized to HNRPM and TBP and log2-transformed. The FGFR2 protein results were normalized to α-tubulin. Each dot represents the results for one fibroblast sample. [file bcr2917-S6.TIFF]
