# Supplementary material for: Allele-specific regulation of FGFR2 expression is cell type-dependent and may increase breast cancer risk through a paracrine stimulus involving FGF10
Source: Breast Cancer Res. 2011 Jul 18;13(4):R72. doi: 10.1186/bcr2917 (PMC3236336; doi:10.1186/bcr2917)
Supplement: Additional file 2 — Supplementary methods. This file contains supplementary methods regarding the quantitative real-time PCR and Western blot analyses. [file bcr2917-S2.DOC]

# Supplementary methods

**Quantitative Real-time PCR**

**Primer design**

Primer pairs for a set of six possible reference genes had been designed and tested previously (Table S3). Three new primer sets were designed for each of our target genes, using the program Beacon Designer. For *FGFR2*, primers were designed to target *FGFR2*-IIIb and IIIc at the same time. Nucleotide Blast (NCBI) was used to test the specificity of each primer set. Primers were manufactured by Sigma-Aldrich. We tested each primer pair in duplicate in a qPCR on the dilution series of the reference sample. The three primer pairs were then compared for PCR efficiency and correlation coefficient as calculated by the program iQ™5 version 2.0 (Bio-Rad Laboratories, Hercules, CA, USA), and the melting curve analysis was used to compare target specificity. Finally, the PCR product was purified and sequenced by Sanger sequencing, which confirmed amplification of the correct target cDNA (data not shown, primers in Table S3).

**Selection of reference genes**

To select reference genes, we performed a qPCR with primer pairs for six potential reference genes on cDNA of six different fibroblast samples, and on cDNA of 14 different epithelial cell cultures. The program GeNorm was then used to select the genes that ranked highest for expression stability[1]. The program selected the two best performing reference genes, *TBP* and *HNRPM*, for the fibroblasts analyses, while for the epithelial cell cultures three reference genes (*TBP*, *HNRPM* and *SRPR*) were selected.

**cDNA**The amount of RNA was determined using the Nanodrop 2000 (Thermo Fisher Scientific, Amsterdam, the Netherlands). cDNA was made by first linearizing 1 µg of RNA in 13.2 µl water for 15 minutes at 65 ºC and then adding 0.5 mM dNTPs, 50 ng of Oligo(dT)_15_ primer (Promega, Leiden, the Netherlands), 500 ng of Random primers (Promega, Leiden, the Netherlands), 20 units of RNase inhibitor (RNasin®, N251A, Promega, Leiden, the Netherlands) and 4 units of the reverse transcriptase enzyme (Reverse Transcriptase AMV, Roche, Woerden, the Netherlands) in a final volume of 20 µl. After incubation at 42 ºC for 1 hour the cDNA was stored at -20ºC.

**qPCR protocol**

cDNA was diluted 20 times and qPCR was carried out in a 25 µl amplification mixture containing 5 µl of the diluted template, 1x iQ SYBR Green Supermix (Bio-Rad Laboratories, Hercules, CA, USA) and 7.5 pmol of the forward primer and the reverse primer. qPCR was performed on the Bio-Rad iCycler thermal cycler, using the following conditions: an initial step of 95ºC for 10 minutes, followed by 40 cycles of 95 ºC for 30 seconds and 60 ºC for 1 minute. Finally, a melting curve analysis was performed by a stepwise temperature increase from 58ºC to 95ºC.

**qPCR validation**

After the reverse transcriptase reaction, genomic DNA contamination of the resulting cDNA was tested using 1:20 diluted cDNA in a PCR with primers located in exon 5 (forward primer: 5’-CAATGTACACATGTAACACCACAAA-3’) and exon 6 (reverse primer: 5’-TGAAACAAACTCCCACATACCA-3’) of *BRCA2*. A PCR product of 59 bp verified the presence of cDNA, while a 180bp product was indicative of residual genomic DNA contamination. RNA isolation was repeated if DNA contamination was present. Furthermore, the forward and reverse primers for several target genes were located in different exons, which would result in an aberrant melting curve if contamination by genomic DNA was present in a cDNA sample during qPCR. A genomic DNA sample was also analyzed in each qPCR to compare the melting curve, but the genomic DNA peak was never found in melting curve analyses of cDNA samples. Therefore DNA contamination of our cDNA samples was very low to non-existent. In each qPCR two negative controls were also present. These never showed replication before cycle 35, and therefore could be considered as negative. We analyzed all melting curves and excluded results when the melting curve showed an aberrant primer-dimer peak that was more than one third of the height of the correct peak. This resulted in rejection of the results for 48 out of a total of 1824 measurements (2.6%). PCR efficiency was always between 90 and 117%. The correlation coefficient of the dilution series was always 0.96 or higher.

**Data analysis**

The qPCR results were analyzed in the program iQ5 (BIO-RAD). The threshold RFU was manually adjusted to the lowest possible value were all samples in a plate showed linear amplification. The program then calculated the threshold cycle (Ct) for each sample. In each qPCR, a dilution series of a reference sample was present in duplicate. By comparing the threshold cycle of each sample to those of the dilution series, the amount of gene expression was calculated relative to the reference sample (the first dilution step of the reference sample was set to 1). We used the base 2 logarithm of these ratios, so that results were normally distributed, a necessity for the statistical tests we performed. All samples were measured at least twice for each gene and results were compared. For all genes except *FGFR2*, the duplicate measurements showed excellent replication. Variation was larger for *FGFR2*, therefore the qPCR for this gene was repeated four times. After calculating the average of these two or four measurements, we normalized the results for the genes of interest by correcting them for the relative amounts of the reference genes. Statistical analyses were performed in PASW Statistics version 17.0. We confirmed normal distribution using the Kruskal-Wallis test. Then, one-way ANOVA was used to compare the mean expression levels of *FGFR2* for the three genotype groups. Spearman’s Rho was used to analyze correlation between the expressions of two genes.

**Western Blot analyses**

**Measuring FGFR2 protein levels**

Total protein was extracted from fibroblast cultures by washing the cells with PBS, followed by adding 400 µl of a solution containing 40 mM Tris-HCl, 3% SDS, 16.5 % glycerol, 0.005% bromophenol blue and 0.05 M DTT. As a positive control, 5 ng of the full-length FGFR2 recombinant protein P01 (Abnova GmbH, Heidelberg, Germany, H00002263-P01) was also diluted in 40 µl of this solution. Subsequently all protein solutions were incubated at 99˚C for 8 minutes and immediately transferred to a 12% acrylamide / bisacrylamide gel (Bio-Rad Laboratories, Hercules, CA, USA). For all analyses, 40 µl of each sample was measured twice on separate gels. The Odyssey protein Molecular Weight Marker (928-40000, LI-COR Biotechnology, Cambridge, UK) was added to each gel to allow estimation of molecular weights. Indirect Western blotting was performed as previously described[2] using the following conditions. Blotting to a nitrocellulose membrane was performed overnight at 4˚C, followed by overnight blocking using 4% non-fat milk powder in PBS. The membranes were exposed to the primary antibodies (Table S4) in 4% non-fat milk powder in PBS-Tween overnight at 4˚C. The membranes were incubated with the fluorescently-labeled secondary antibodies (Table S4) in a solution of 4% milk powder and 0.01% SDS in PBS-Tween, for 1 hour at room temperature, protected from light. The Odyssey® Infrared Imaging System and the Odyssey 3.0 software (LI-COR Biotechnology, Cambridge, UK) were used to visualize and identify the bands. The integrated intensities of the bands were determined and corrected for background signal (lane background). Alpha-tubulin was measured in each lane, and its integrated intensity was used to correct for input differences in each lane.

**Measuring phosphorylation of downstream targets of FGFR2**

To analyze phosphorylation of downstream targets of FGFR2, total protein was isolated from the 6-well plates by removing the medium and adding 250 µl of a solution containing 50 mM Tris-HCl, 5% β-mercaptoethanol, 2% SDS, 0.5% bromophenol blue and 30% glycerol to the wells, after which the solutions were incubated at 99˚C for 3 minutes and immediately used in western blot analysis. This analysis was performed in a manner similar to that described to measure FGFR2 protein levels, except PVDF membranes were used, blocking time was reduced to 2 hours and exposure time to the primary antibodies (Table S4) to 1 hour at room temperature.
